# Minimally Invasive Surgery for Pediatric Adrenal Masses—Report on Four Cases

**DOI:** 10.1055/s-0039-1694058

**Published:** 2019-10-31

**Authors:** Ahmed ElHaddad, Christoph Castellani, Erich Sorantin, Martin Benesch, Eva Kampelmühler, Georg Singer, Holger Till

**Affiliations:** 1 Department of Paediatric and Adolescent Surgery, Medical University of Graz, Graz, Austria; 2 Department of Radiology, Division of Pediatric Radiology, Medical University of Graz, Graz, Styria, Austria; 3 Department of Paediatrics and Adolescent Medicine, Medical University of Graz, Graz, Austria; 4 Institute of Pathology, Medical University of Graz, Graz, Austria

**Keywords:** adrenal, tumor, oncology, laparoscopy

## Abstract

The dignity of adrenal masses in children varies from benign lesions like adenoma and ganglioneuroma to malignant tumors like adrenocortical carcinoma and neuroblastoma. Any surgical approach, especially minimally invasive surgery (MIS), requires careful risk stratification based on oncological and technical criteria. Herein, we present four patients who underwent MIS for adrenal masses. Laboratory testing differentiated between simple cysts and adenoma, but could not identify a child with adrenocortical tumor preoperatively. Analysis of image-defined risk factors excluded vascular encasement in all cases. All patients underwent laparoscopic adrenalectomy without complications. Histopathology revealed simple cyst, ganglioneuroblastoma, adenoma, and potentially malignant adrenocortical tumor in one patient/case each. All specimen showed clear margins and no recurrence was noted at a mean follow-up of 18 months.

## Introduction

The adrenal gland consists of a medulla and a cortex and may give rise to various types of tumors. While adenomas and carcinomas may develop in the cortex, the medulla may be the origin of neuroblastomas, pheochromocytomas, and ganglioneuromas. All of these lesions may require surgical intervention by an open or a minimally invasive approach. The risks and benefits of pediatric laparoscopic adrenalectomy for malignant or semibenign lesions remain a matter of vivid discussion. While simple adrenal cysts or adenomas are benign lesions and a minimally invasive approach may be considered, adrenocortical carcinomas and (ganglio)neuroblastomas require careful assessment of risk factors such as serum biomarkers, hormonal status, and image-defined risk factors (IDRFs) . 1 2 3 In the present case series, distinct characteristics of technical feasibility and oncological safety of laparoscopic approaches for pediatric adrenal masses are discussed.

## Case Presentation

### Case 1

**Fig. 1:**
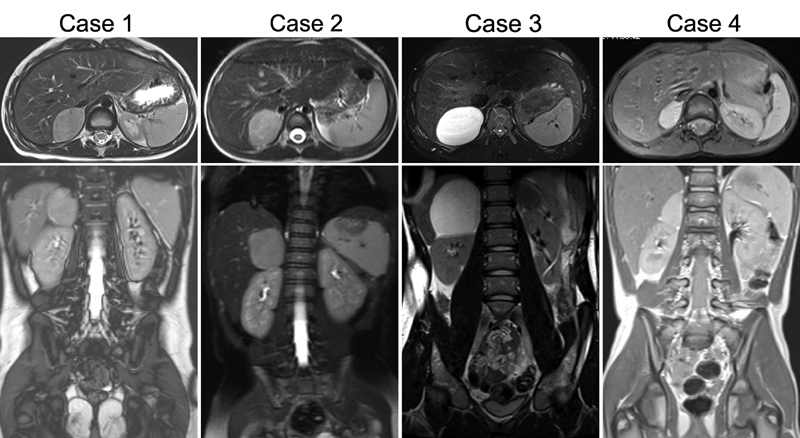
Magnetic resonance imaging of all four patients. None of them presented with image-defined risk factors.

A 3-year-old boy presented with precocious puberty (tanner stages P2 and G2). X-rays of the extremities showed an accelerated bone age of ∼6 years. Ultrasound (US) and magnetic resonance imaging (MRI) of the abdomen revealed a right adrenal mass of approximately 4.0 × 2.5 × 4.0 cm ( Fig. 1 ). The laboratory workup of the patient is displayed in Table 1 . Laparoscopic adrenalectomy was performed. Histopathological examination confirmed complete resection of an adrenal adenoma with hamartoma components. At the 2-year follow-up, the patient presented with a normalized hormone status (cortisol, testosterone, dehydroepiandrostendionsulfate, androstenedione, 17-hydroxyprogesterone, adrenocorticotropic hormone) and without signs of recurrence in the US.

**Table 1:** Laboratory workup of the patient

Parameter	Range	Case 1	Case 2	Case 3	Case 4
Age at surgery		3 years	1,5 years	15 years	7 years
Body weight (kg)		18 (90–97th percentile)	13 (97th percentile)	71 (80th percentile)	20 (3rd percentile)
Height (cm)		103 (90th percentile)	82 (97th percentile)	182 (88th percentile)	121 (10th percentile)
Serum LDH (U/L)	120–340	218	367 (+)	193	248
Serum NSE (mg/mL)	0–26	26.0	33.1 (+)		38 (+)
VMA urine (µg/mg creatinine)	<10	9.0	8.1		5.0
HVA urine (µg/mg creatinine)	<15	15.0	11.0		10.0
Dopamine urine (µg/mg creatinine)	<0.85	0.68	0.64		0.52
Bone marrow aspiration					Negative
N-Myc amplification					Negative
LH (mU/mL)	1.0–14.0	0.22 (−)	<0.1 (−)	1.94	
FSH (mU/mL)	0.89–11.72	0.59 (−)	<0.1 (−)	1.80	
Basal hGH (mU/mL)	0.5–3.0	6.9 (+)	2.1		
PRL (ng/mL)	2.1–29.2		14.0		
IGF-1 (mU/mL)	30.0–300.0	120.6			
Basal ACTH (pg/mL)	10–51		56.6 (+)	17.1	
Basal cortisol (ng/mL)	43–220		5.8 (−)		
Androstenedione (ng/mL)	1.0–14.0	3.3		2.58	
DHEA-S (µg/mL)	0.39–4.63	8.1 (+)		1.62	
Total Testosterone (ng/mL)	2.41–8.30	0.4	0.77	2.88	
17OH-Progesterone (ng/mL)	0.2–0.9	0.88		0.66	
Histology		Adrenal adenoma	Potentially malignant ACT	Simple adrenal cyst	Ganglioneuroblastoma

Abbreviations: ACT, adrenocortical tumor(+)-elevated; (−) - decreased; ACTH, adrenocorticotropic hormone; DHEA-S, dehydroepiandrostendionsulfate; FSH, follicle stimulating hormone; hGH, human growth hormone; HVA, homovanillylic acid; IGF, insulin-like growth factor; LDH, lactate dehydrogenase; LH, luteinizing hormone; NSE, neuron specific enolase; PRL, prolactin; VMA, vanillylmandelic acid.

### Case 2

A one- and half-year-old female presented with a precocious puberty (virilization with clitoris hypertrophy and pubarche P2–3). Laboratory parameters are shown in Table 1 . Abdominal US showed a hypoechogenic lesion in the right adrenal gland. MRI ( Fig. 1 ) revealed a largely solid, relatively sharply delimited, contrast-enhancing lesion of the right adrenal gland (4.0 × 3.0 × 3.5 cm) without IDRFs. Thus, a transperitoneal laparoscopic adrenalectomy was performed. Histopathology revealed a large, encapsulated adrenocortical tumor (ACT) with a diameter of 5 cm and malignant potential with tumor-free resection margins. At the 11-month follow-up, there were no signs of recurrence.

### Case 3

A 15-year-old male patient presented with exercise-triggered recurrent right abdominal pain. US and MRI demonstrated an uncomplicated cystic structure in the right adrenal gland with sharp delineation to the upper renal pole and without pathological contrast enhancement ( Fig. 1 ) giving no indication for surgery. In his routine US checkups, the size of the cyst increased to 8.0 × 7.6 × 5.8 cm over 2 years indicating surgical removal. Additional information about laboratory workup is given in Table 1 . Laparoscopic adrenalectomy was done and histopathology revealed a mesothelial cyst. At the 12-month follow-up, there were no sonographic signs of recurrence of the cyst.

### Case 4

A 7-year-old boy presented due to recurrent abdominal pain. The routine US revealed an inhomogeneous expansion of approximately 3 × 3 × 2 cm with calcifications in the right adrenal gland. Laboratory data are displayed in Table 1 . MRI showed a well circumscribed slightly inhomogeneous mass in the right adrenal gland of approximately 3.3 × 2.6 × 2.0 cm. Bone marrow biopsy was normal but metaiodobenzylguanidine (MIBG) scan showed pathologically increased tracer uptake. Anterior laparoscopic adrenalectomy was done with complete tumor resection. Histopathological examination revealed clinical stage I maturing ganglioneuroblastoma with free margins. No recurrence was observed at 2-year follow-up.

## Discussion

In this case series, we present four pediatric patients with a broad spectrum of the adrenal gland masses undergoing laparoscopic resection.

Generally, tumors of the adrenal gland can be malignant (with descending incidence: neuroblastoma, adrenocortical carcinoma, or sarcoma) or benign (with descending incidence: adenoma, ganglioneuroma, cysts, or adrenal hyperplasia). 3 In infants, tumors have to be discriminated from postpartum adrenal hematomas as an important differential diagnosis that has to be considered in the diagnostic workup. Regarding tumors, pheochromocytomas are rare adrenal masses that can be benignant or malignant in their behavior. When selecting patients for a minimally invasive approach, special care has to be taken to identify these children who potentially benefit from minimally invasive surgery (MIS) (better cosmetic results, less pain, and shorter hospital stay 4 5 ) and to exclude those who might be harmed by this procedure (risk of inadequate resection, tumor recurrence, trocar site metastasis, tumor rupture, and spillage as well as tumor growth or dissemination in relation to CO _2_ insufflation 6 7 ).

Guidelines for adrenalectomy poorly define the indications for MIS in the pediatric age group. The rarity of adrenal lesions in this age group, a wide pathologic spectrum, the predominance of malignant lesions, large dimensions of the masses in relation to small body size of the patients, and technical challenges with miniature instruments may be hypothesized as the underlying reasons. 8

After initial suspicion, patients with adrenal masses undergo routine diagnostic workups usually involve imaging (US, computed tomography and/or MRI) paired with laboratory analysis (routine blood chemistry, differential blood count, hormone profiles, and tumor markers). Concerning imaging special emphasis is put on the presence or absence of IDRFs. Infiltration of the renal pedicle, encasement of major vessels (inferior vena cava, aorta, superior mesenteric artery, celiac trunk, iliac, and hypogastric vessels), the porta hepatis, dumbbell tumors, muscular infiltration, and compression of kidney or ureter have been identified as IDRFs. 1 If present, IDRFs may point to malignant behavior of the tumor. Furthermore, complete resection could be achieved in only 26% of cases presenting with IDRFs in a study of Günther et al. 1

While MIS is the mainstay in benign lesions, 3 it has to be discussed critically in malignant tumors. In selected cases of neuroblastoma (size ≤ 5 cm diameter and absence of vascular encasement), MIS led to an equal long-term oncologic outcome compared with open surgery. 9 Similarly, de Lagausie et al state that sizes greater than 6 cm, enlarged veins, and involved adjacent organs or vessels are relative contraindications for laparoscopic adrenalectomy for neuroblastomas. 10 Overall, the International Pediatric Endoscopic Group 2010 guidelines state that there are no absolute contraindications for laparoscopic adrenalectomy as long as the principles of cancer surgery are maintained. 11

Special care has to be taken to identify the rare case of a malignant ACT. Suspicion should be raised in case of adrenal masses presenting with IDRFs and especially in patients with signs of precocious puberty. ACTs have a high risk of intraoperative rupture (∼20% of cases) with consecutive tumor spillage. 12 Recent recommendations opted against laparoscopic biopsy or resection of tumors suspicious for or known to be ACT even when MIS appears feasible and tempting, because complete resection of the mass is the prerequisite for recurrence-free survival. 13 14 However, there are also reports of successful MIS in ACTs. 8 15 16 These reports are supported by the current German AWMF recommendations that advocate that a MIS approach in ACTs is feasible as long as the principles of oncological surgery are maintained. 17 Furthermore, a recent publication of adult ACT cases found evidence that the open and laparoscopic approach may be comparable in terms of recurrence-free survival for patients with stage I and II ACT when the principles of surgical oncology are respected. 18

Taken the recent literature into account, the indication for MIS was clear in cases 1, 3, and 4 presented in this series. Histology of case 2 revealed a potentially malignant adrenocortical tumor raising the question, if MIS was the optimal therapy for this patient. As there was no evidence of IDRFs and the tumor size was < 5 cm, MIS was performed. Although an open procedure may have been beneficial, the tumor could be removed completely by MIS and the patient is free of recurrences until now (although the follow-up interval of 11 months is relatively low for an oncologic patient).

In conclusion, MIS is indicated in benign lesions, but has to be discussed critically in case of potential malignancy. In these cases, a MIS approach may be considered in selected patients without IDRFs as long as the principles of oncologic surgery can be maintained.

## Notes

New Insights and the Importance for the Pediatric Surgeon In this series of four cases distinct characteristics of technical feasibility and oncological safety of laparoscopic approaches for pediatric adrenal masses are discussed.
